# PSMA-PET und „radio-guided surgery“ bei zervikaler Lymphadenektomie

**DOI:** 10.1007/s00106-022-01197-3

**Published:** 2022-07-13

**Authors:** Julia P. Lingl, Felix Böhm, Thomas Wiegel, Ambros J. Beer, Thomas K. Hoffmann

**Affiliations:** 1 Klinik für Hals-Nasen-Ohrenheilkunde, Kopf- und Halschirurgie, Universitätsklinikum Ulm, Frauensteige 12, 89075 Ulm, Deutschland; 2 Klinik für Strahlentherapie und Radioonkologie, Universitätsklinikum Ulm, Albert-Einstein-Allee 23, 89081 Ulm, Deutschland; 3 Klinik für Nuklearmedizin, Universitätsklinikum Ulm, Albert-Einstein-Allee 23, 89081 Ulm, Deutschland; 4 Surgical Oncology Ulm, i2SOUL Consortium, Albert-Einstein-Allee 23, 89081 Ulm, Deutschland

**Keywords:** Prostakarzinom, Halslymphknotenmetastase, Zervikale Lymphknotenextirpation, Prostata spezifisches Antigen, Gamma-Sonden-unterstützte Chirurgie, Prostate cancer, Cervical lymph node metastasis, Cervical lymph node exstirpation, Prostate specific membrane antigen, Gamma probe assisted surgery

## Abstract

Wir berichten über einen 75 Jahre alten Patienten mit suspekten linksseitig zervikalen Lymphknoten in Region IV. Detektiert wurden diese in der PSMA-PET-MRT (Prostataspezifisches Membranantigen-Positronenemissions-Magnetresonanztomographie) zum Restaging bei bekanntem Prostatakarzinom mit laborchemischem PSA(Prostataspezifisches Antigen)-Anstieg in der onkologischen Nachsorgeuntersuchung. Zur histologischen Sicherung wurde eine hochselektive Lymphadenektomie in der linksseitigen Region IV unter γ‑Sonden-Kontrolle nach ^99m^Tc(Technetium-99m)-PSMA-Markierung durchgeführt. Hierbei wurden 2 vergrößerte Lymphknoten mit deutlicher Traceraufnahme entfernt. Die histopathologische Untersuchung ergab die Diagnose von Lymphknotenmetastasen des bekannten Prostatakarzinoms. Mithilfe der „radio-guided surgery“ können unter Verwendung eines adäquaten Tracers supraselektiv pathologische Lymphknoten im Kopf-Hals-Bereich detektiert und sanierend entnommen werden.

## Abstract (en)

A 75-year-old male patient with suspicious cervical lymph nodes in level IV on the left side is presented. The cervical mass was detected in PSMA-PET-MRI as part of a restaging examination conducted due to an increase of PSA levels in the context of the patient’s known prostate cancer. We conducted a selective cervical level IV lymphadenectomy with the aid of a gamma probe subsequent to radiolabelling with ^99m^Tc-PSMA. Two visibly enlarged lymph nodes with high gamma probe signals could be extracted. Histopathological examination revealed lymph node metastases of the known prostate cancer. Using an adequate tracer radio-guided surgery helps to detect pathological lymph nodes in the head and neck region allowing for supraselective resection.

## Anamnese

Die Vorstellung des 75-jährigen Patienten erfolgte mit suspekten linksseitig zervikalen Lymphknoten in Region IV/V. 2008 wurde ein Prostataadenokarzinom diagnostiziert, bei initialem TNM-Stadium von pT3b pN1(1/4) cM0 mit Gleason-Score = 7 (4 + 3). Es erfolgte primär eine radikale Prostatektomie (R0) mit pelviner Lymphadenektomie sowie eine adjuvante Radiotherapie der Primärtumorregion und Lymphabflusswege gefolgt von einer Androgendeprivationstherapie. Bei paraaortaler/infrarenaler Lymphknotenmetastasierung erfolgte 2016 eine PSMA-radio-guided-salvage-Lymphadenektomie (PSMA = prostataspezifisches Membranantigen) sowie eine erneute Radiatio der Paraaortalregion.

Die suspekte zervikale Lymphadenopathie ist im Rahmen des Restagings mittels PET-MRT bei simultan laborchemischem PSA-Anstieg (0,7 ng/ml) detektiert worden. Klinisch war der Patient im HNO-ärztlichen und urologischen Untersuchungsbereich beschwerdefrei.

## Befund

**Figure 1:**
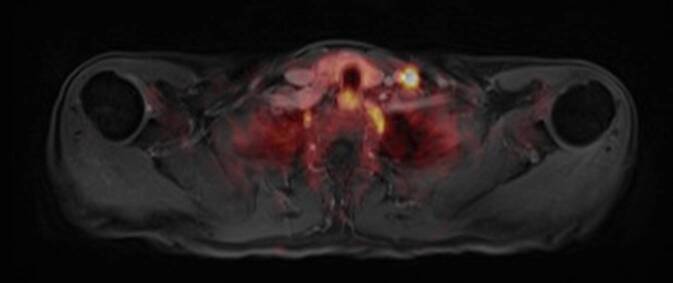

Im PSMA-PET-MRT zeigten sich 2 größenprogrediente, PSMA-spezifisch anreichernde Lymphknoten linksseitig tief zervikal in Region IV (Abb. 1). Ansonsten bestand kein Anhalt für Fernmetastasierung oder ein lokoregionäres Rezidiv.

**Figure 2:**
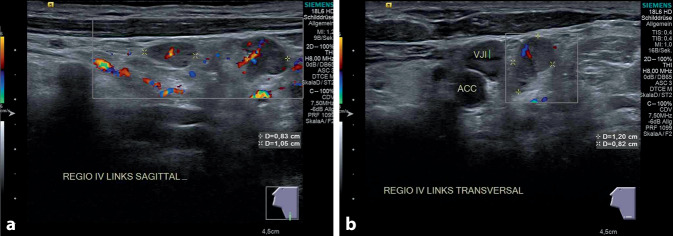

In der insgesamt blanden HNO-ärztlichen Routineuntersuchung waren zervikal keine pathologischen Lymphknoten palpabel. In der B‑Bild-Sonographie (Abb. 2) konnten in Region IV/V links 2 inhomogene lymphknotenähnliche Strukturen, rundlich konfiguriert mit einem maximalen Durchmesser von 1,2 cm, jeweils mit einem Solbiati-Index < 2, aber erhaltenem hilären Perfusionsmuster, dargestellt werden.

Sowohl die klinische Untersuchung als auch die nuklearmedizinische Bildgebung ergaben keinen Anhalt für einen Primarius i.B.d. HNO-Fachgebiets.

## Diagnose

Bei PSMA-spezifisch anreichernden Lymphknoten mit simultanem PSA-Anstieg sowie fehlendem Anhalt für einen Primarius der Kopf-Hals-Region ergab sich der Verdacht auf zervikale Metastasen des bekannten Prostatakarzinoms.

Auf Grundlage der PSMA-Spezifität kommen differenzialdiagnostisch Metastasen eines Plattenepithelkarzinoms, eines adenoidzystischen Karzinoms oder eines Schilddrüsenkarzinoms infrage [2].

## Therapie und Verlauf

Gemäß interdisziplinärem Tumorboardbeschluss erfolgte eine Lymphadenektomie des Levels IV links unter γ‑Sonden-Kontrolle nach vorheriger Radionuklidmarkierung mit ^99m^Technetium-PSMA. Intraoperativ stellten sich i.B.d. linken Venenwinkels 2 vergrößerte Lymphknoten dar, die entfernt wurden. Sowohl im Operationssitus wie auch extrakorporal zeigte sich unter Verwendung der Tracersonde ein deutlich positives Signal der resezierten Knoten. Im Bereich der Resektionshöhle war post excisionem kein Tracersignal mehr zu detektieren. Der weitere peri- und postoperative Verlauf gestaltete sich regelrecht.

Histopathologisch zeigten sich beide Lymphknoten durchbaut von einem vorwiegend cribriform gebauten Adenokarzinom. Die größere der beiden Metastasen wies eine Infiltration des perinodalen Fettgewebes (ENE+) auf. In der Immunhistochemie waren Zytokeratin-8/-18 und PSMA kräftig positiv. Die α‑Methylacyl-CoA-Racemase (AMACR) und der Androgenhormonrezeptor waren lediglich fokal positiv. Somit bestätigte sich die Diagnose von Lymphknotenmetastasen des Prostatakarzinoms.

Postoperativ sank der PSA-Wert mit 0,056 ng/ml annähernd gegen Null, sodass seitens der Urologie auf eine adjuvante Therapie zugunsten einer Active Surveillance verzichtet wurde.

## Diskussion

Zervikale Lymphknoten sind ein häufiger Manifestationsort für Metastasen maligner Tumoren der Kopf-Hals-Region, in der Majorität der Fälle bei einem Primarius eines Plattenepithelkarzinoms des oberen aerodigestiven Trakts oder der Haut. Insgesamt weisen 1 % aller Halslymphknotenmetastasen einen extrazervikalen Primärtumor auf. Am häufigsten ursächlich sind Primarien der Lunge, der Niere und der Mamma [4].

Prostatakarzinome wachsen lokal destruierend und bis zu 70 % der Patienten weisen bei Erstdiagnose eine Tumorausdehnung über die Organkapsel hinaus auf. Die Tumoren metastasieren vorwiegend lymphatisch in die pelvinen und paraaortalen Lymphknotenstationen, ossär in die Wirbelsäule und Röhrenknochen und parenchymatös in Lunge, Blase und Leber [9]. Eine Metastasierung in supraklavikuläre Lymphknoten wird bei weniger als 0,5 % der Patienten berichtet [3, 9].

Als ursächlich für die Entwicklung von Halslymphknotenmetastasen wird eine hämatogene Metastasierung über das venöse System der Wirbelsäule oder über den Batson-Venenplexus diskutiert [1], alternativ, aber weniger wahrscheinlich erscheint eine lymphatische Metastasierung durch einen retrograden Fluss von Lymphe aus dem Ductus thoracicus in die zervikalen Lymphbahnen [4].

Einen neuen Ansatz zur gezielten Resektion von Lymphknotenmetastasen des Prostatakarzinoms stellt die „PSMA-radio-guided surgery“ dar. PSMA, ein transmembranes Glykoprotein, ist in Prostatakarzinomzellen stark hochreguliert [2]. Für die „PSMA-radio-guided surgery“ erhalten betroffene Patienten präoperativ i.v. einen radioaktiven PSMA-Liganden, der in der Zielläsion, also dem karzinominfiltrierten Lymphknoten, anreichert und so eine intraoperative Detektion mittels γ‑Sonde erlaubt [8].

Für die „PSMA-radio-guided surgery“ von abdominellen und pelvinen Lymphknotenmetastasen bei Prostatakarzinomrezidiven existieren bereits vielversprechende Ergebnisse bezüglich der Metastasendetektion [6, 7]. Maurer et al. konnten intraoperativ zwischen tumorinfiltrierten und gesunden Lymphknoten unterscheiden (Sensitivität 83,6 %; Spezifität 100 %), gefolgt von einer vollständigen Entfernung aller im präoperativen PSMA-PET detektierten Läsionen [5].

In dieser Kasuistik berichten wir über den seltenen Fall eines Patienten mit zervikaler Metastasierung eines Prostatakarzinoms sowie über eine moderne Technik, um Prostatakarzinommetastasen intraoperativ zu identifizieren. Nach ausgiebiger systematischer Literaturrecherche scheint dies der erste in der Literatur beschriebene Fall einer „PSMA-radio-guided surgery“ in der Lymphadenektomie von Halslymphknoten zu sein. Die Identifikation der Metastasen war problemlos, schnell und zielsicher möglich. Intra- oder postoperative Komplikationen traten nicht auf. Der postoperative Abfall des PSA-Werts in den Nullbereich weist auf eine vollständige Entfernung der karzinominfiltrierten Lymphknoten mittels der verwendeten Technik hin.

Obwohl Halslymphknotenmetastasen eine ungewöhnliche Manifestation eines Prostatakarzinoms sind, stellen sie eine wichtige Differenzialdiagnose insbesondere bei der hohen Prävalenz des Prostatakarzinoms in der männlichen Bevölkerung dar. Eine „PSMA-radio-guided surgery“ ist bei Patienten mit Prostatakarzinom neben der Resektion von abdominopelvinen Lymphknoten auch bei Verdacht auf eine zervikale Metastasierung eine sinnvolle Option. Sie erscheint vorteilhaft in der exakten Lokalisierung und Resektion insbesondere bei kleinen Metastasen in Anbetracht der großen Gesamtzahl an zervikalen Lymphknoten.

## Fazit für die Praxis

- Halslymphknoten stellen einen ungewöhnlichen Manifestationsort von Metastasen eines Prostatakarzinoms dar, sollten aber bei positiver Anamnese für ein Prostatakarzinom in der Patientenvorgeschichte als Differenzialdiagnose berücksichtigt werden.
- Prostatakarzinome können auch Jahre nach Erstdiagnose und multiplen Vortherapien noch in ungewöhnliche Regionen metastasieren. Regelmäßige PSA-Wert-Kontrollen sind ein wichtiges Hilfsmittel zur frühzeitigen Indikationsstellung erweiterter, kostenintensiver Diagnostik. Ein entsprechender Marker ist für Kopf-Hals-Tumorpatienten mit Plattenepithelkarzinomen bisher leider nicht verfügbar.
- Die „PSMA-radio-guided surgery“ ist technisch auch bei V. a. zervikale Metastasierung umsetzbar und bringt Vorteile bei der exakten Lokalisierung und Resektion von kleinen metastatischen Läsionen.

## Abkürzungen

99mTc: Technetium-99m
AMACR: α‑Methylacyl-CoA-Racemase
ENE: Extranodale Extension
MRT: Magnetresonanztomographie
PET: Positronenemissionstomographie
PSA: Prostataspezifisches Antigen
PSMA: Prostataspezifisches Membranantigen
